# Patch frequencies in rhombic Penrose tilings

**DOI:** 10.1107/S2053273323004990

**Published:** 2023-07-24

**Authors:** Jan Mazáč

**Affiliations:** a Fakultät für Mathematik, Universität Bielefeld, Postfach 100131, Bielefeld 33501, Germany; Ateneo de Manila University, Philippines

**Keywords:** patch frequency, tiling, dualization method

## Abstract

This exposition presents an efficient algorithm for an exact calculation of patch frequencies for rhombic Penrose tilings. A construction of Penrose tilings via dualization is recalled and, by extending the known method for obtaining vertex configurations, the desired algorithm is obtained. It is then used to determine the frequencies of several particularly large patches which appear in the literature. An analogous approach works for a particular class of tilings and this is also explained in detail for the Ammann–Beenker tiling.

## Abstract 2

An algorithm is presented for an exact calculation of patch frequencies for a family of tilings which can be obtained via dualization.

## 1. Introduction

The idea of a non-periodic tiling of a plane with fivefold symmetry goes back to Kepler’s famous Figure Aa (Kepler, 1940 ▸). The (rhombic) tiling introduced by Roger Penrose (1974 ▸) is an aperiodic fivefold symmetric tiling of a plane with two prototiles – a thick and a thin rhombus. There are many ways to generate this tiling. One can define local matching rules, or one can think of it as an inflation tiling and define inflation rules. A more algebraic approach is due to de Bruijn (1981*a* ▸,*b* ▸). It relies on the dualization of a pentagrid, *i.e.* the union of five rotated lattices. An overview of the methods can be found in, for example, Baake & Grimm (2013 ▸). Here, we are interested in another algebraic, yet different, approach. It profits from the geometry of the root lattice *A*_4_ and the fact that this lattice is a ‘minimal’ one with fivefold symmetry. Again, this approach uses dualization, in this scenario the duality relation between Voronoi and Delone cells (and their complexes).

Recently, the rhombic Penrose tiling was considered as an infinite graph and it has been studied using tools from graph theory. One can consider its graph-theoretic properties like Hamiltonicity, Eulericity or (perfect) matchings (Flicker *et al.*, 2020 ▸; Lloyd *et al.*, 2022 ▸), but one can also assign an operator acting on this graph and study its spectral properties. Damanik *et al.* (2022 ▸) studied the properties of a Laplacian on various tilings, among them the rhombic Penrose one. They studied a tile model for the Laplacian and they were able to show some examples of locally supported eigenfunctions, which are also known from other reports (Fujiwara *et al.*, 1988 ▸). Recently, Oktel published several papers dealing with a similar problem for the vertex model for different tilings (Oktel, 2021 ▸, 2022 ▸; Keskiner & Oktel, 2022 ▸). For all these models, one can further study the integrated density of states (IDS), which is a function that counts the number of states (different eigenfunctions) up to a given energy. It is known that this function is discontinuous. More precisely, if one can find a locally supported eigenfunction with energy *E* of the Laplacian, the IDS has a discontinuity jump at point *E*. The size of this gap is at least as big as the frequency of the eigenfunction’s support, *i.e.* the frequency of the corresponding patch (Damanik *et al.*, 2022 ▸). Thus, knowing the frequency, one obtains a lower bound on the size of the gap. Damanik and co-workers used a direct approach to calculate the frequencies, namely, they counted the number of occurrences of the support of a given eigenfunction in growing approximants of the entire tiling. The same method was employed earlier by Fujiwara *et al.* (1988 ▸). There is an obvious disadvantage to this method in that one has to deal with the boundary of the approximants, which may include parts of the studied patch. Another problem constitutes the way of choosing the approximants. Lastly, the resulting frequency is always given as a numerical approximation. Therefore, we aim to fill this gap by showing an algebraic way of obtaining the frequencies of arbitrary finite patches in (not only) Penrose rhombic tilings exactly, without any need for the inflation method. For Penrose rhombic tilings, there already exists a method introduced by Zobetz & Preislinger (1990 ▸) using de Bruijn’s approach, which enables a calculation of the frequencies of vertex configurations in generalized Penrose tilings. Still, our approach provides a more general framework as it allows us effectively to calculate an exact frequency of arbitrary large patches for a wider class of tilings. As far as we are aware, no algorithm yet exists that would actually enable the calculation of exact frequencies for arbitrary finite patches.

This paper is structured as follows. In Section 2, we recall the geometry of the *A*_4_ lattice and its Voronoi complex and of their dual objects. Further, in Section 3, we recall a representation of a cyclic group of order 5 (which acts naturally on the lattice *A*_4_), which exhibits fivefold symmetry in a plane. Section 4 evokes the dualization method and its benefits. These sections are almost fully based on the work of Baake *et al.* (1990 ▸). We recall these concepts as they are necessary for the algorithm. The crucial point is that it describes tilings rather than point sets by a variant of the projection method known as dualization. In particular, the standard model set approach via the intersection of translated windows (Baake & Grimm, 2013 ▸, Cor. 7.3) is in practice unable to give the frequencies of large patches. The algorithm for determining the frequencies is presented in Section 5. In Appendix *A* [App appa], we apply it to several patches coming from the work of Damanik *et al.* (2022 ▸). Appendix *B* [App appb] is devoted to a brief summary of the patch frequencies in Ammann–Beenker tilings.

## 2. The root lattice *A*_4_, its dual and their properties

The lattice *A*_4_ can be understood in different ways. Perhaps the most natural one (explaining its name) is that *A*_4_ is the root lattice of the semisimple Lie algebra 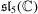 . On the other hand, its explicit description as an intersection of the primitive five-dimensional cubic lattice with a four-dimensional (4D) hyperplane allows us to simplify some calculations. Thus, let **e**_1_, …, **e**_5_ be the standard basis vectors of 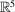 and set 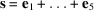 . Further, let 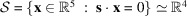 be a four-dimensional hyperplane in 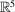 . Then, one has 

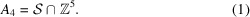

The resulting lattice is generated by four vectors, namely 

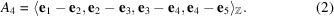

**Figure 1:**
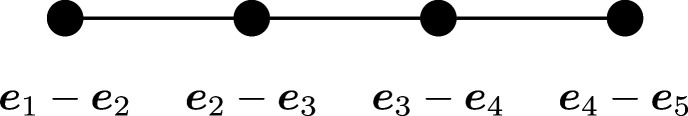
The Dynkin diagram *A*_4_. Every node represents a basis vector and their geometry is encoded via the lines. If two vertices are connected, their scalar product is −1. Otherwise, they are orthogonal.

Alternatively, we can depict the root lattice *A*_4_ as a Dynkin diagram (Fig. 1 ▸).

Note that the generating vectors **e**_*i*_ − **e**_*i*+1_ are fundamental (or simple) roots of the root system of 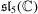 . This system consists of 20 root vectors, namely **e**_*i*_ − **e**_*j*_ with 1 ≤ *i*, *j* ≤ 5 and *i* ≠ *j*. For our further analysis, we need to describe the maximal point symmetry group 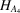 at the origin of the lattice *A*_4_. It is isomorphic with the automorphism group of the generating root system. The root system is, by definition, invariant under the action of the Weyl group *W*(*A*_4_), which is the permutation group *S*_5_ in this case. Moreover, central inversion is an additional symmetry generating the group *Z*_2_. Thus, the group 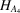 is isomorphic to 

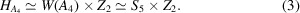

The 20 root vectors also determine the Voronoi cell 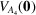 around the origin, *i.e.* all vectors in the underlying hyperplane 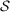 which are not further away (with respect to the Euclidean distance) from the origin than from any other lattice point, so 

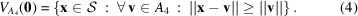

The Voronoi cell can also be understood as an intersection of closed half-spaces 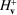 corresponding to **v** ∈ *A*_4_ defined as 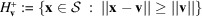 . Here, the Voronoi cell 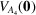 is fully determined by the 20 root vectors, *i.e.* one has 

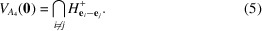




To obtain a more explicit description of the Voronoi cell 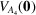 , we have to employ the dual lattice 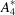 and its fundamental domain. The dual lattice can be obtained in many ways. Following Conway’s approach via glue vectors (Conway & Sloane, 1999 ▸), one has 

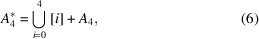

with the glue vectors 

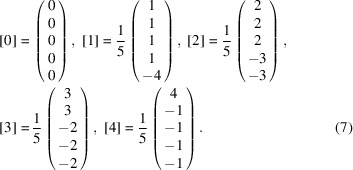




This description allows one immediately to recognize *A*_4_ as a proper sublattice in its dual lattice 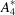 . Moreover, the quotient group 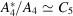 is of order 5 and the representatives can be chosen as the glue vectors. On the other hand, for upcoming calculations, it is convenient to write down the generators of the lattice. Here, 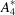 is spanned by the vectors 

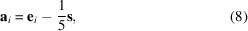

with 1 ≤ *i* ≤ 5 and **s** as above. Note that the generating vectors are not linearly independent since 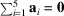 . Finally, one can use them to describe the Voronoi cell, 

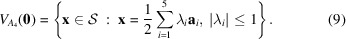

This object is a regular four-dimensional convex polytope, sometimes considered as a dual polytope to the runcinated 5-cell. It has full symmetry *W*(*A*_4_) × *Z*_2_. The polytope possesses 30 vertices, 70 bounding edges and 60 bounding polygons (*i.e.* polytopes of dimension two), and 20 bounding polytopes of dimension three. Henceforth, we refer to them as *k*-boundaries, with 0 ≤ *k* ≤ 3. Baake *et al.* (1990 ▸) provided a careful analysis of all *k*-boundaries and their explicit description, together with one of their corresponding duals in the sense of Kramer & Schlottmann (1989 ▸). Important to us here are the 2-boundaries, the vertices and the corresponding dual objects as follows.

The 2-boundary polygons are given by 

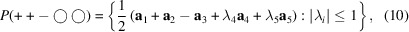

together with all polygons obtained via vertex permutations and sign flips. There is an explicit action of the group 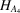 on the set of 2-boundaries. This action can be encoded on the level of the signature 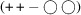 as well. In particular, a permutation just permutes the indices, a sign flip affects the signs and 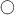 remains unchanged. From the geometric point of view, 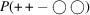 is a rhombus, and therefore it will play a crucial role in constructing the Penrose rhombus tiling. The 2-boundary dual to 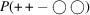 is the triangle 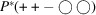 defined as 

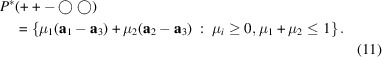

The correspondence between *P* and P* is one to one and the boundaries intersect with their duals at precisely one point.

The 30 vertex points of the Voronoi cell 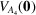 are exactly those points of 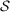 with the largest distance to the lattice *A*_4_. In terms of the theory of root lattices, they are called holes (Conway & Sloane, 1999 ▸). Points with the maximum possible distance to *A*_4_ are called deep holes and the remaining ones are shallow holes. In our case, the vertex 

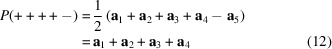

and all its images under *W*(*A*_4_) × *Z*_2_ are the shallow holes, whereas the 20 points of type 

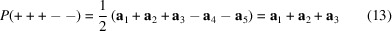

are the deep holes.

The dual objects to deep and shallow holes are four-dimensional cells. Namely, one obtains a four-dimensional simplex 

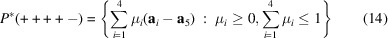

and a four-dimensional Archimedean polytope 

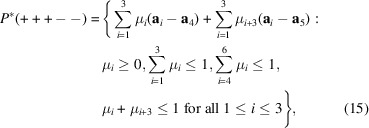

and all their images under the symmetry operations 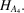


Since *A*_4_ is a lattice, one has the same vertex configuration around any of its points up to translation. Thus, the Voronoi cell 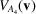 around **v** is a translation 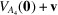 . Further, one can collect all *k*-boundaries and think of them in terms of complexes. In particular, one can define the Voronoi complex 

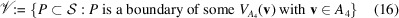

and for 0 ≤ *k* ≤ 4 its *k*-skeleton 

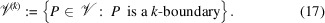




The properties of the duality lead to the dual Voronoi complex and its dual *k*-skeleton as, respectively, 

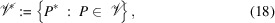




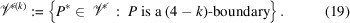




Taking any vertex v* of the Voronoi cell 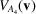 for some **v** ∈ *A*_4_, *i.e.* 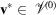 , the associated dual object, which is a full 4D polytope, will be denoted by V*(v*) as it plays a similar role to the Voronoi cell.

As mentioned above, different points appear within the point sets studied. We have to deal with points of the lattice *A*_4_ and with the vertices of its Voronoi cells. The latter are split into two categories, deep and shallow holes. In order to distinguish them, one can introduce a modulo function *r* defined for any point 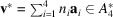 as 

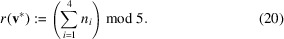

It is clear that 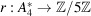 . Since the generating vectors **e**_*i*_ − **e**_*i*+1_ of the lattice *A*_4_ fulfil 

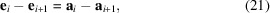

one immediately has 

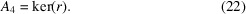

Further, one obtains the characterization of shallow and deep holes in terms of *r*(v*). In particular, 

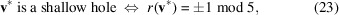




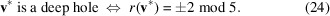

Remark 2.1 The function *r* corresponds to the index function in de Bruijn’s construction (de Bruijn, 1981*a* ▸,*b* ▸). This is not surprising because de Bruijn’s construction implicitly uses the root lattice as a Minkowski embedding of fifth roots of unity, as explained by Baake & Grimm (2013 ▸, Section 7.5.2).

## 3. Representation with fivefold symmetry

We have already mentioned that *W*(*A*_4_) acts on the generators of *A*_4_ via permutations of the basis vectors **e**_*i*_. This action has two invariant subspaces, namely 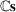 and 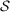 . The linear representation of *S*_5_ ≃ *W*(*A*_4_) is irreducible on 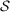 , and to find a real irreducible representation capturing the fivefold symmetry in plane one has to restrict oneself to a suitable subgroup. Therefore, consider the cyclic group *C*_5_, a subgroup of *W*(*A*_4_). Its generating element *g* = (12345) acts on the basis (**e**_1_, …, **e**_5_) via the matrix 

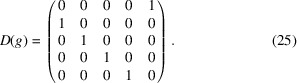

To find the possible representations means to find the real Jordan form of *D*(*g*) via an orthogonal matrix *J*. The real Jordan form reads 

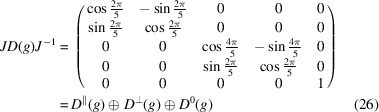

and provides three irreducible real representations 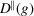 , 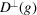 and 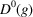 . The matrix *J* read columnwise provides a new basis, as one can directly read from 

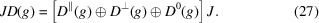

In particular, one has 

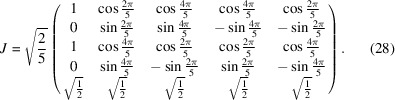




Since the trivial representation *D* ^(0)^(*g*) is carried by the subspace 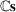 , it follows that *D* ^∥^(*g*) and *D* ^⊥^(*g*) are contained in 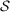 . Thus, one has to decompose 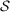 as a direct sum of two subspaces, 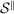 and 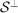 . The representations of *g* in 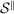 and 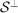 are rotations about 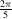 and 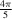 , respectively.

**Figure 2:**
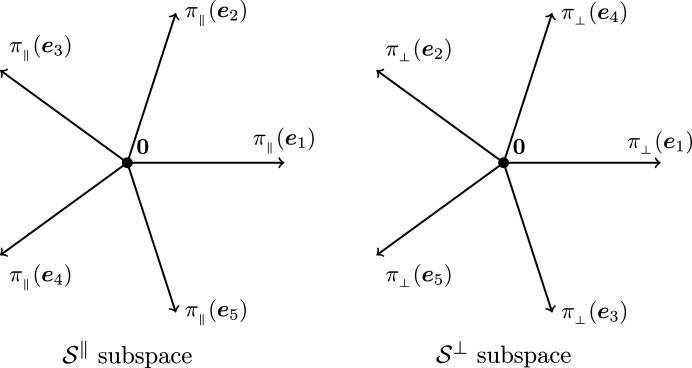
Projections of the standard basis **e**_1_, …, **e**_5_ into the two subspaces 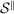 and 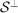 .

Denote by π_∥_ and π_⊥_ the projections from 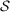 onto 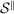 and 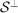 , respectively. The projections of basis vectors π_∥_(**e**_*i*_) are given by the first and second rows of the *i*th column of *J*, and those of π_⊥_(**e**_*i*_) are given by the third and fourth rows of the same column. Fig. 2 ▸ depicts the projections of the basis vectors **e**_*i*_, which exhibit the desired fivefold symmetry. Since 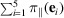 = 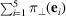 = **0**, one immediately has π_∥_(**a**_*i*_) = π_∥_(**e**_*i*_) and π_⊥_(**a**_*i*_) = π_⊥_(**e**_*i*_) for all 1 ≤ *i* ≤ 5.

**Figure 3:**
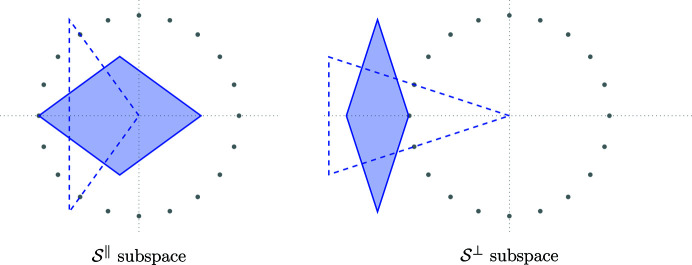
Images of the 2-boundary 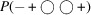 and its dual 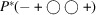 under the projections π_∥_ and π_⊥_. The solid blue rhombi correspond to projections of the 2-boundary, whereas the dashed line indicates the projection of its dual. The grey points are the 20th roots of unity scaled by 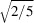 .

Projecting the 2-boundary *P* and its dual 2-boundary P* in both spaces results in a set of triangles and rhombi, which we use later for the construction of the Penrose tiling. Fig. 3 ▸ shows the projections of 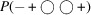 and 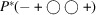 . Note that the rhombus vertices always consist of one projection of a shallow hole and three projections of deep holes. The position of the shallow hole will be needed later to distinguish different patterns.

## 4. Dualization method

One can obtain a space tiling via the *dualization method*. This method was described in detail by Kramer & Schlottmann (1989 ▸), and Baake & Grimm (2013 ▸) provided an illustrative overview. To employ this method, one needs a Voronoi complex 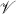 , its dual (Delone) complex 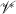 and a suitable cutting plane, which carries the desired tiling. To obtain a non-periodic tiling, one has to choose the cutting plane so that it contains at most one lattice point.

**Figure 4:**
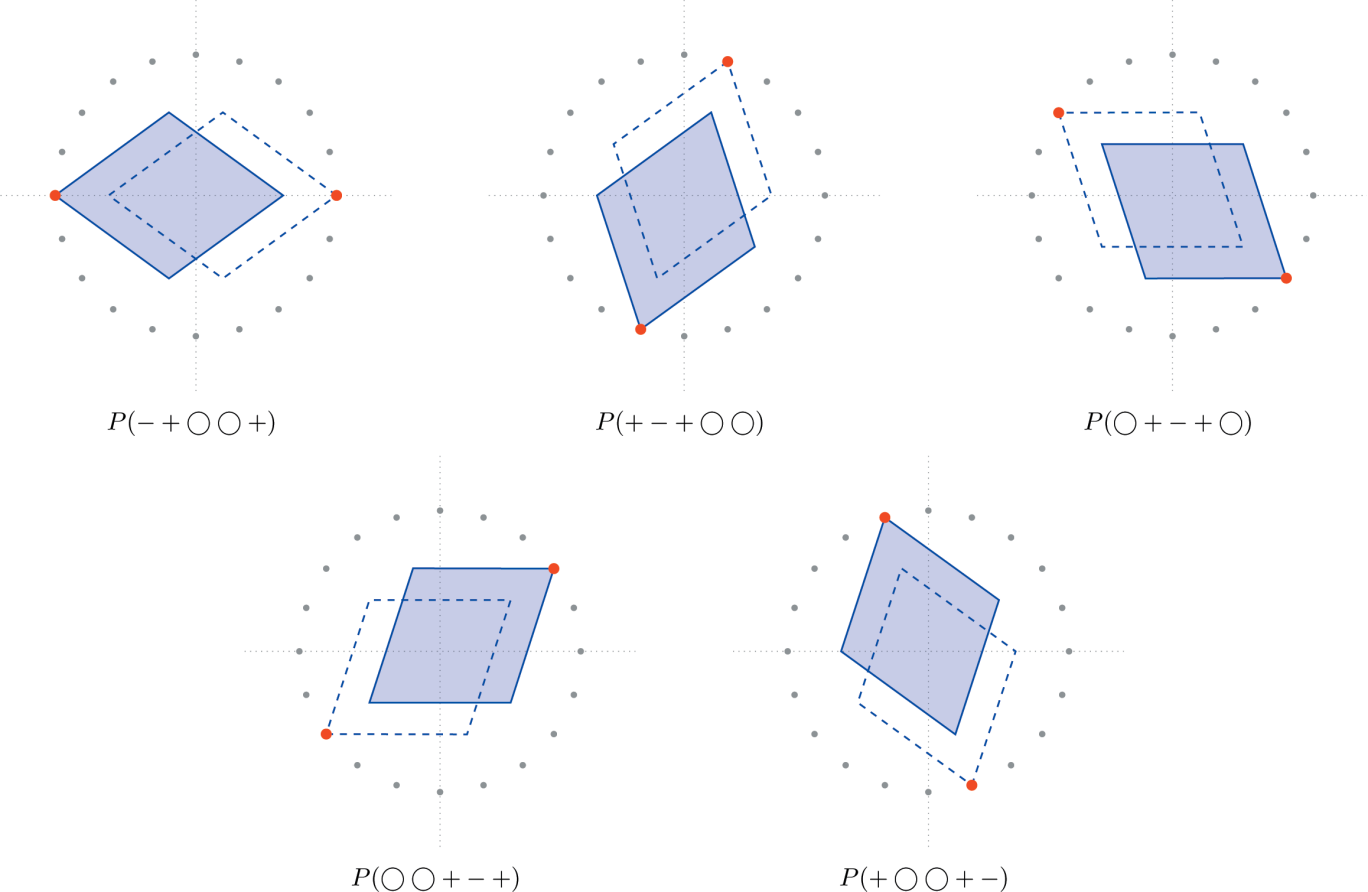
Projections of the different (modulo translation) 2-boundaries *P* in 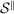 which result in a thick rhombus. The solid rhombi correspond to the labels, whereas the dashed rhombi are their space inversions. The red point attached to a given rhombus indicates the shallow hole. The grey points are the 20th roots of unity scaled by a factor 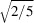 .

The construction works in general as follows. Whenever the cutting plane intersects a *k*-boundary of the Voronoi complex, the dual (4 − *k*)-boundary is projected onto the cutting plane. In our case, we wish to obtain the rhombic Penrose tiling. Therefore, we restrict ourselves to the skeletons 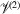 and 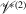 . Fig. 4 ▸ shows the projections of the different (modulo translation) 2-boundaries onto the 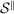 , which are the thick Penrose rhombi.

We choose as the cutting plane a translation of 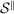 by a vector 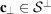 . To ensure aperiodicity, we have to choose **c**_⊥_ such that it is not contained in any π_⊥_ projection of any 1-boundary of 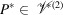 ; see Baake *et al.* (1990 ▸) for further details. The vector **c**_⊥_ restricts the elements of 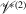 which one projects onto 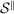 , since the cutting plane 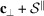 intersects a 2-boundary *P* if and only if π_⊥_(P*) contains **c**_⊥_. The resulting tiling (which depends on the choice of **c**_⊥_) can be described as 

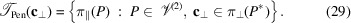




The vertices of 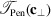 are projections of the vertex points of certain Voronoi domains *V*(**v**) for some **v** ∈ *A*_4_. As already discussed above, these vertices are elements of 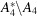 and are of four translation types, as characterized by the function *r*. The vertex points **v** ^*^ are split into four orbits with respect to the translation action of *A*_4_. For each orbit, one can choose a representative 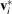 , for example 

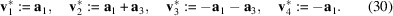

From the construction of 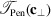 , we see that a point 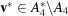 is a pre-image of a vertex point in 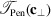 if and only if v* ∈ *P* with **c**_⊥_ ∈ π_⊥_(P*) for some 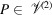 . Note that if a point is an element of a *k*-boundary, the dual (4 − *k*)-boundary lies in the dual cell of that point and vice versa. So v* ∈ *P* if and only if 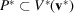 , with V*(v*) being a translation of a dual four-dimensional cell of the form (14) or (15). Thus, π_∥_(v*) is a vertex in 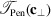 if and only if **c**_⊥_ ∈ π_⊥_(V*(v*)). Two points 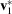 and 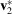 with 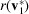 = 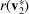 can only differ by a lattice vector. The choice of representatives (30) allows us to relate any point v* with one of them. Define 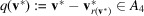 for any v*. Since 

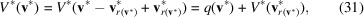

one has 

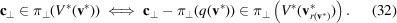

This allows us to rewrite the set of vertex points of 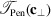 as 

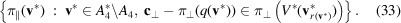




**Figure 5:**
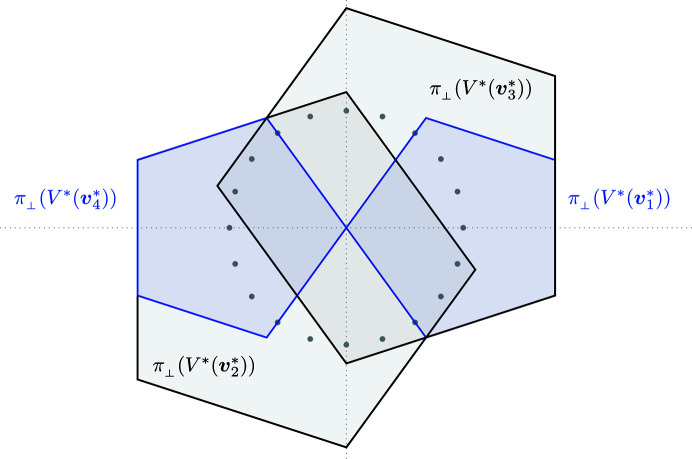
Projections 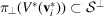 corresponding to the windows. The blue pentagons carry the π_⊥_-projections of shallow holes, whereas the black ones comprise the projections of deep holes. Note that for every window there exists its own lattice. Thus, even though there is a non-trivial intersection of windows, the resulting points must differ, as one expects. The grey points are the 20th roots of unity scaled by a factor 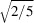 .

This description shows that the set of vertices can be understood as four cut-and-project sets with lattices 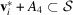 and windows 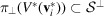 , 1 ≤ *i* ≤ 4. Fig. 5 ▸ shows all four windows 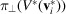 in 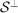 ; for more detail see Example 7.11 and Remark 7.8 in Baake & Grimm (2013 ▸).

Once we have established the description of all vertices of 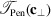 , we can further determine a *vertex configuration* of each vertex, *i.e.* all tiles in 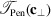 surrounding the vertex π_∥_(v*). The description (29) provides us with a characterization of the tiles surrounding π_∥_(v*). Indeed, a tile π_∥_(*P*) belongs to a vertex configuration of π_∥_(v*) if and only if 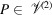 , v* ∈ *P* and **c**_⊥_ ∈ π_⊥_(P*). The problem of finding a vertex configuration around an arbitrary vertex point can be reduced using translation symmetry. We can restrict ourselves to finding all vertex configurations around a representative of each translation class, *i.e.* around the points 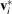 . We can then rewrite the conditions above as 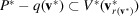 and **c**_⊥_ − π_⊥_(*q*(v*)) ∈ π_⊥_(P*) − π_⊥_(*q*(v*)). So *P* belongs to a vertex configuration of a point v* if and only if the translation of its dual P* by *q*(v*) is a 2-boundary of the dual cell 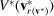 . This gives an algorithm for obtaining the complete vertex configuration around the vertex π_∥_(v*) as follows.

(1) Find all 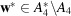 such that 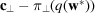 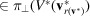 .

(2) For all w* found in Step (1), take the 2-boundary P* of the dual cell 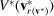 with **c**_⊥_ − π_⊥_(*q*(w*)) ∈ π_⊥_(P*). Then, π_∥_(w*) + π_∥_(*P*) is a tile around π_∥_(v*).

**Figure 6:**
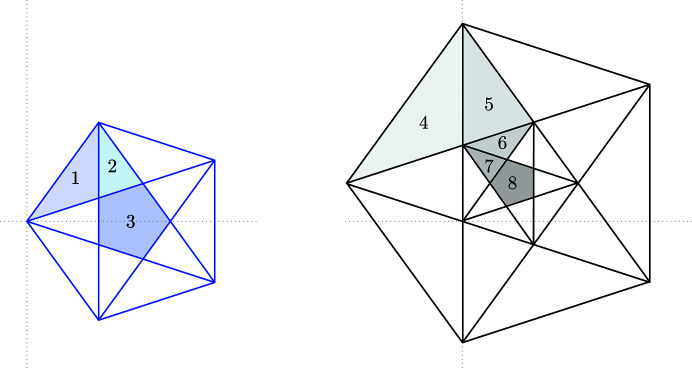
Subdivision of 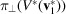 (blue) and 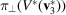 (black) into elementary polygons. The eight possible vertex configurations (modulo rotation by 2π/5 and space inversion) correspond to eight distinct elementary polygons.

**Figure 7:**
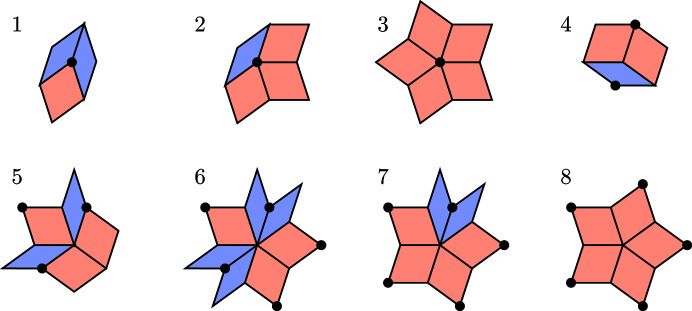
All possible vertex configurations (up to rotation by 2π/5 and space inversion) which are in one-to-one correspondence with the elementary polygons in Fig. 6 ▸. The black points indicate the positions of shallow holes.

We choose **c**_⊥_ so that **c**_⊥_ − π_⊥_(*q*(w*)) lies in the interior of π_⊥_(P*). This is a crucial observation. It forces all tiles π_∥_(w*) + π_∥_(*P*) belonging to a particular vertex configuration to have, at the level of π_⊥_(P*), an overlap in the 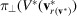 . We can use this property to determine and characterize all possible vertex configurations with respect to translations in 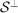 as follows: a set 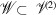 of 2-boundaries is a valid vertex configuration of a vertex of type *i* if and only if 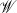 is maximal with respect to the property that 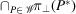 is non-empty. The projection of 2-boundaries of the dual cells 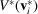 divides the 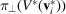 into convex polygons, called *elementary polygons* (Baake *et al.*, 1990 ▸). They have pairwise distinct interiors, each representing a distinct vertex configuration (and vice versa). Fig. 6 ▸ shows the elementary polygons for 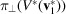 and 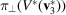 . The corresponding vertex configurations are shown in Fig. 7 ▸.

The choice of the cutting plane ensures that the projections of the vertices of a valid infinite Penrose tiling onto 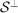 are dense and uniformly distributed. Thus, we can use them to determine the frequencies of the vertex configurations via the areas of the elementary polygons. Denote by *E* an elementary polygon. The relative frequency 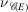 of vertex configuration 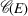 corresponding to the elementary polygon *E* is then given by 

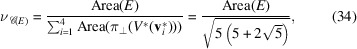


*i.e.* by exactly the fraction of the total area of windows it occupies. We list the frequencies of all vertex configurations in Table 1 ▸. We include the frequency of the given patch as well as the cumulative frequency of all patches of the same type, *i.e.* all patches that lie in the same orbit under the rotation and space inversion.

The sum of all total frequencies equals one, and thus we have a consistency check. Since Penrose tiling defines a strictly ergodic dynamic system (in the usual way) (Robinson, 1996 ▸), we conclude that there are no other vertex configurations. If there were any others, they would come with a strictly positive measure, which is the patch frequency.

Recall that the *frequency module* of a tiling space 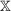 (in our case, the tiling space generated by rhombic Penrose tilings) is the minimal 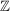 -module 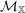 that contains all frequencies of finite patches of the tiling. Here, we obtain the following specific result.

Proposition 4.1 The frequency module 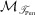 of the Penrose tiling is 

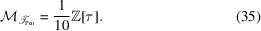

Proof Consider any patch of the Penrose tiling. We can always find an 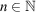 such that this given patch is contained in a level-*n* supertile of some vertex configuration 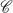 . Since the Penrose tiling is an inflation/deflation tiling, its level-*n* supertiles around a given vertex configuration are equivalent to the original vertex configuration scaled by a factor τ^*n*^. Therefore, the supertile itself has a frequency given by 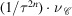 . The factor 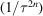 comes from the observation that the frequency is inversely proportional to the area. Since τ is a unit in 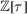 , so is τ^2*n*^. Thus, to determine the frequency module, it suffices to consider the 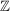 -module generated by 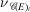 , 1 ≤ *i* ≤ 8, *i.e.*


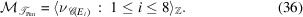

Since 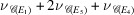 = 1/10 and 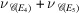 = 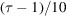 , one has 

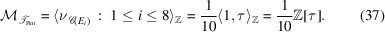

□

## 5. General patch frequencies and their calculation

The idea behind the above construction can be extended to any patch in Penrose rhombic tilings. Choose a vertex of a tile and relate all tiles of the patch to this vertex. One has to be careful and distinguish consistently between deep and shallow holes. One then obtains a list of all tiles and their relative positions with respect to the chosen central tile. By transitioning into 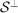 , one obtains a list of all dual triangles and their relative distance. Their intersection determines the frequency of the patch in the same way as in the case of vertex configuration. This intersection is always a convex polygon (since one intersects a finite number of triangles) and its area can be computed easily. Note that some minimal subset of the triangles entirely determines this intersection and working only with them can increase the computational speed considerably.

**Figure 8:**
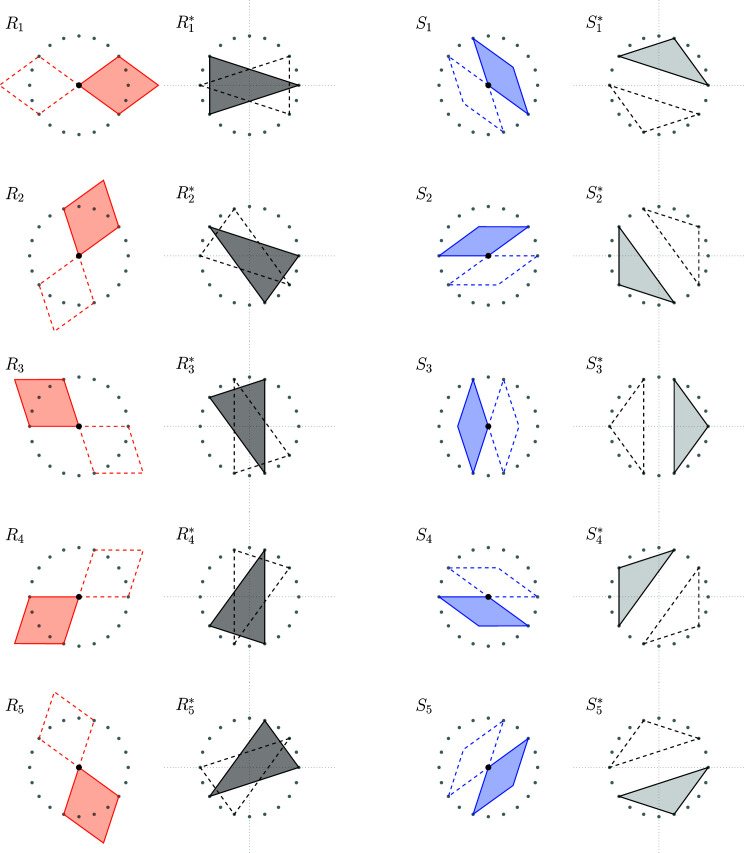
A list of all possible tiles (with respect to their orientations and placement of a shallow hole) in rhombic Penrose tilings and their duals in 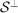 . Tiles are depicted relative to the shallow hole. The exact correspondence between a tile in the list and a projection of a 2-boundary is, for example, the following. If a tile of type *R*_1_ corresponds to 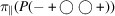 , the dual triangle 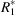 *is equal* to 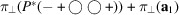 , *i.e.* we capture its actual position in ⊥-space. Fixing the positions of the duals allows us to work in coordinates relative to a given point, the ‘origin’. Everything is then shifted by a suitable vector representing the relative distances of objects from the origin.

Let us list, in Fig. 8 ▸, all possible tiles together with the shallow holes attached to each of them. We place them so that the shallow hole indicates the ‘origin’ relative to the given tile. More precisely, we depict them in coordinates which are translated by the shallow vertex of a given tile. We also include the dual tile and its projection in 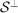 . The projection is also centred on the relative origin. There is an extra advantage to such a choice, namely, the vertices of dual triangles in 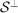 are placed at the 20th roots of unity scaled by the factor 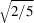 . Since the frequency is given by a ratio of two areas, the scaling factor does not play a role. This allows a precise calculation, simply by employing a suitable subfield of 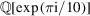 . In fact, one can work with integer coefficients.

We can now describe the algorithm that allows us to determine the frequency of a given patch. We start with an arbitrary finite patch of the Penrose tiling.

(1) Detect all shallow holes in the patch. This can be done via the allowed vertex configurations.

(2) Identify the type of each tile in the patch as *R*_*i*_, *S*_*i*_ or their space inversions (*RI*_*i*_, *SI*_*i*_) from the list given in Fig. 8 ▸.

(3) Choose any shallow hole, the ‘origin’, from the vertices of the patch and fix it.

(4) Make a list of all positions of all tiles (their shallow holes) relative to the origin. Since the edges of the rhombi are projections π_∥_(**a**_*i*_), the resulting position vector can always be written as 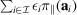 , where 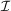 parametrizes the path on the edges from the origin to the desired point and ε_*i*_ ∈ {±1} denotes the orientation of the vectors π_∥_(**a**_*i*_) in the path.

(5) Apply the dual correspondence, *i.e.* to each translated tile 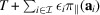 assign the dual 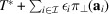 , with *T* ∈ {*R*_*i*_, *S*_*i*_, *RI*_*i*_, *SI*_*i*_}.

(6) Find an intersection of all 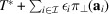 from the list. This can be done via any clipping algorithm, for example the Sutherland–Hodgman algorithm (Sutherland & Hodgman, 1974 ▸).

(7) Calculate the area of the intersection.

(8) Divide the area of the intersection by the total area of the windows, *i.e.* by 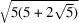 . This yields the relative frequency.

Note that one can choose any clipping algorithm since one has to deal with triangles only (for different lattices one obtains general convex polygons). Under this condition, most clipping algorithms are sufficiently robust. Moreover, at least the Sutherland–Hodgman algorithm ensures that the resulting coordinates of the vertices of the intersection are contained in the same field as the coordinates of the polygons, since each step of the algorithm relies on solving systems of two linear equations with the coefficients being the coordinates of the vertices of the polygons.

Finally, computing the area of a polygon determined by its vertices can be done via the shoelace formula (or Gauss’s area formula) (Koecher & Krieg, 2007 ▸), which is also within the field.

**Figure 9:**
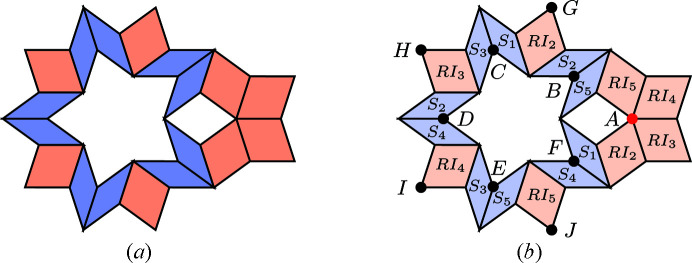
(*a*) The original plain diamond ring patch with 18 tiles. (*b*) The same patch after the identification process with indicated shallow holes (dots) and with a chosen origin (red dot). The tiles are labelled with respect to the shallow hole they contain and the picture shows the situation after Step 3 of the algorithm.

**Figure 10:**
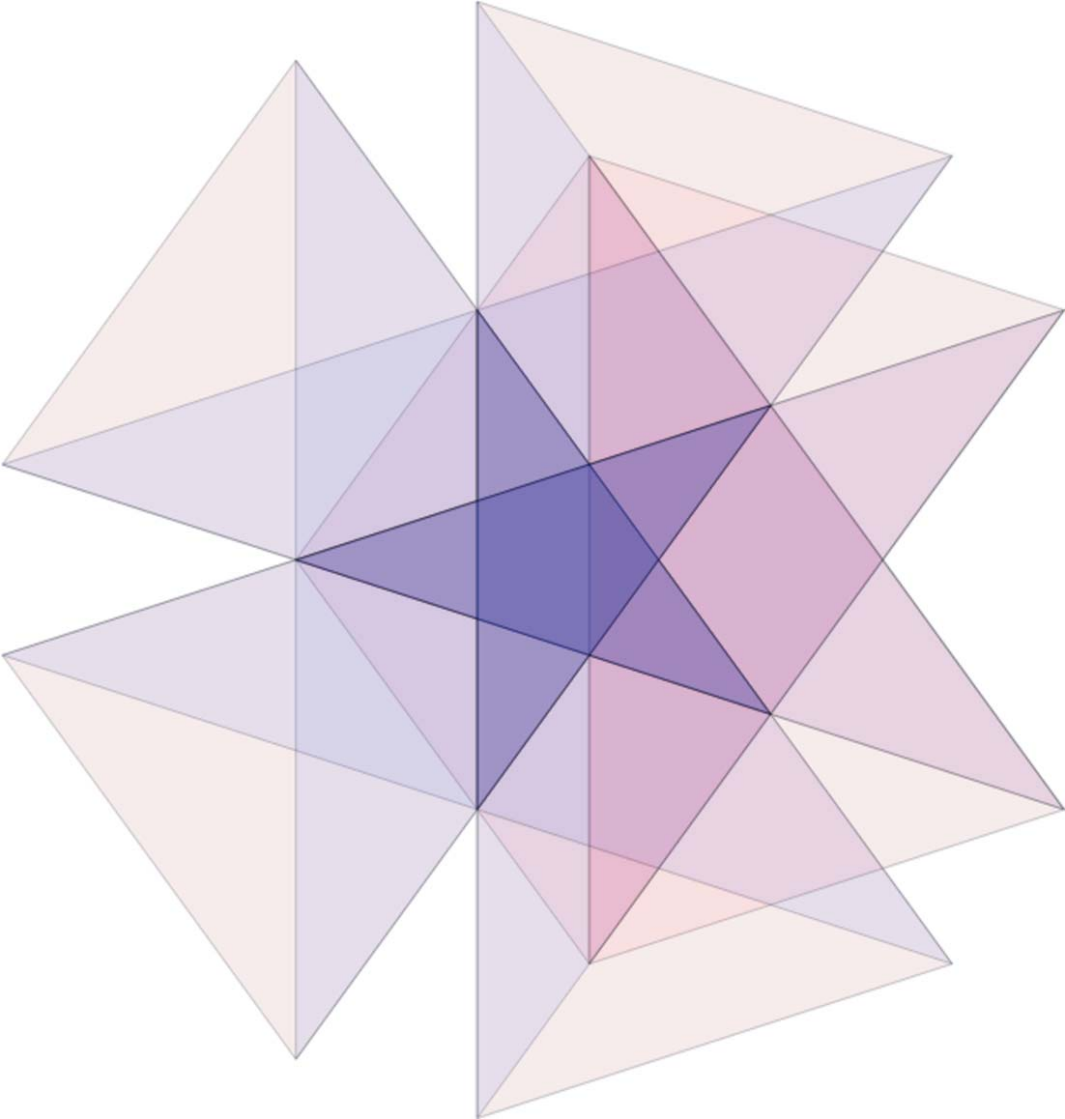
The intersection of dual tiles of the diamond ring patch. They possess a common intersection, the small violet triangle.

Let us demonstrate the procedure on the following patch [this patch, called a *diamond ring*, supports an eigenfunction of a discrete Laplacian on the Penrose tiling; see Damanik *et al.* (2022 ▸) for further details]. Fig. 9 ▸(*a*) shows the initial data of the algorithm. Fig. 9 ▸(*b*) shows the results of Step 2 (determining the shallow holes) and Step 3 (labelling the tiles). Table 2 ▸ summarizes the paths from the origin (red point *A*) to the (black) shallow holes (labelled with letters *B* to *J*), *i.e.* the relative translation vectors, *i.e.* the result of Step 4. Finally, Fig. 10 ▸ shows the result of the correspondence described in Step 5, *i.e.* it depicts the corresponding dual triangles in 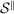 and their intersection (Step 6), which is, in this particular case, a triangle. Its area (Step 7) is 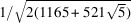 . Thus, the frequency of the diamond ring patch reads 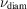 = 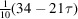 = 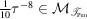 . The total frequency of this patch (*i.e.* of all its possible rotations and space inversions) is 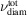 = 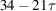 . We include other patches mentioned by Damanik *et al.* (2022 ▸) in Appendix *A* [App appa].

The algorithm for obtaining patch frequencies can also be used for an entire class of tilings, namely, for those tilings obtained via the dualization method. Usually, there is no need to distinguish between deep and shallow holes, which makes the procedure slightly easier. On the other hand, another restriction may occur, but the idea and the basic scheme remain the same. By interchanging the roles of triangles and rhombi, one can obtain the Tübingen Triangle Tiling (TTT) (Baake *et al.*, 1990 ▸). Using a different root lattice, one can also obtain patch frequencies for a plethora of quasiperiodic tilings with eight- and 12-fold symmetry, including the Ammann–Beenker tiling (Baake & Joseph, 1990 ▸; Baake *et al.*, 1991 ▸). Further details are given in Appendix *B* [App appb].

## Figures and Tables

**Figure 11:**
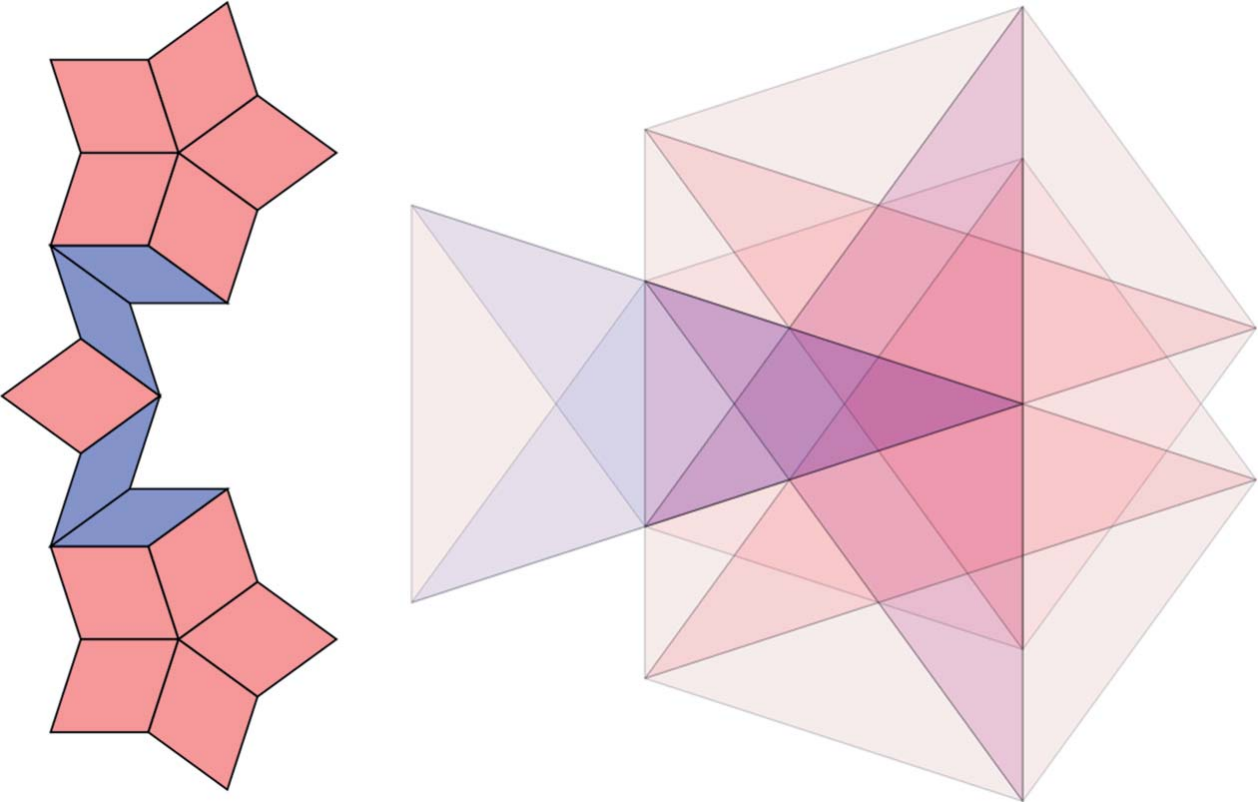
The two star patch with 15 tiles. Its frequency is 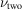 = 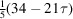 = 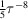 .

**Figure 12:**
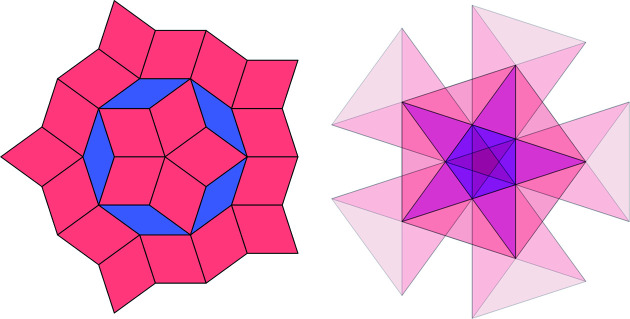
The filled circle patch with 25 tiles. Its frequency is 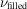 = 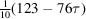 = 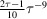 .

**Figure 13:**
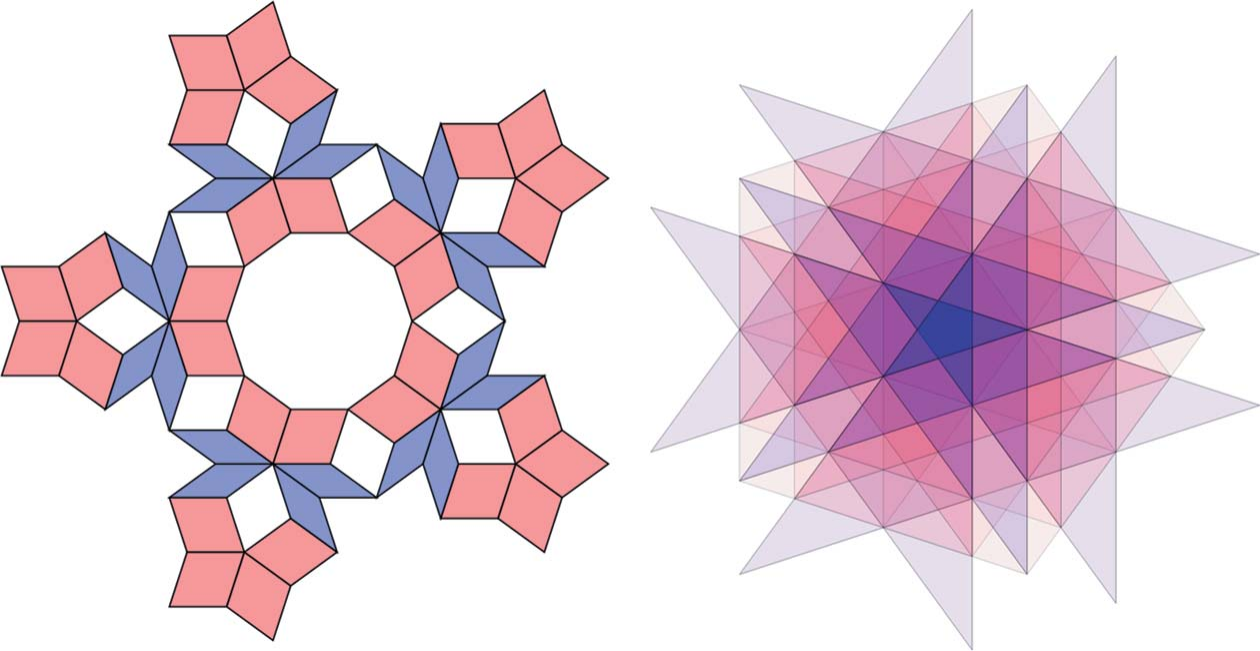
The big star patch with 50 tiles. Its frequency is 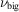 = 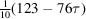 = 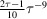 .

**Figure 14:**
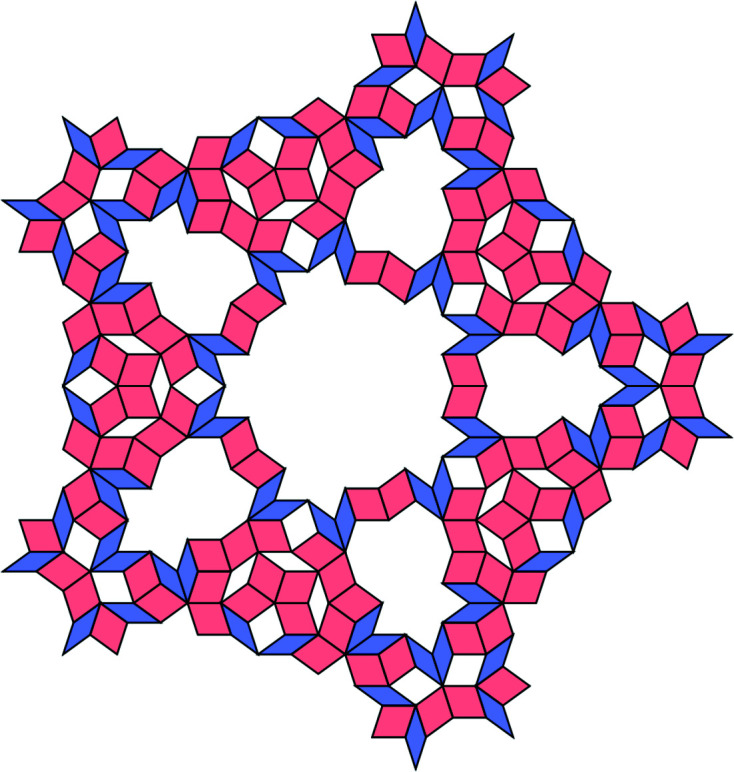
A 200-tile patch.

**Figure 15:**
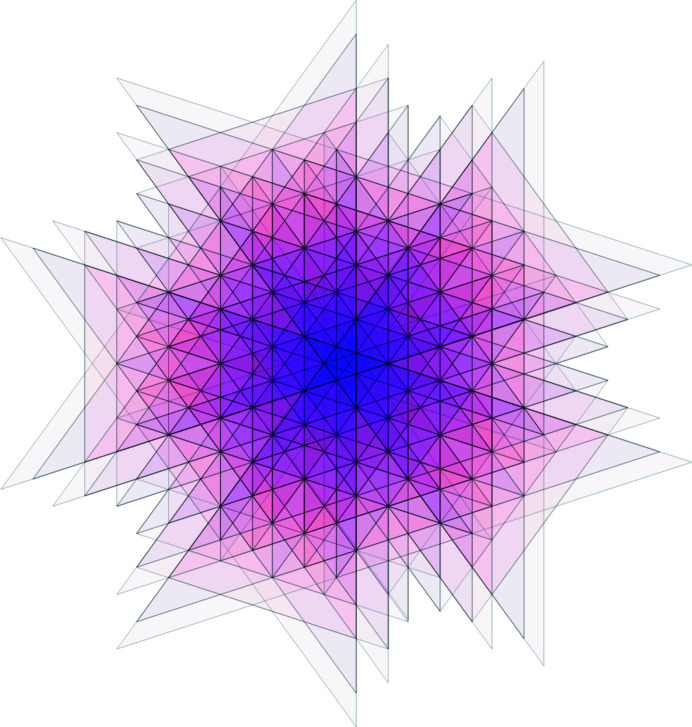
The dual image of the patch from Fig. 14. The frequency of this patch is 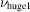 = 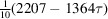 = 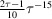 .

**Figure 16:**
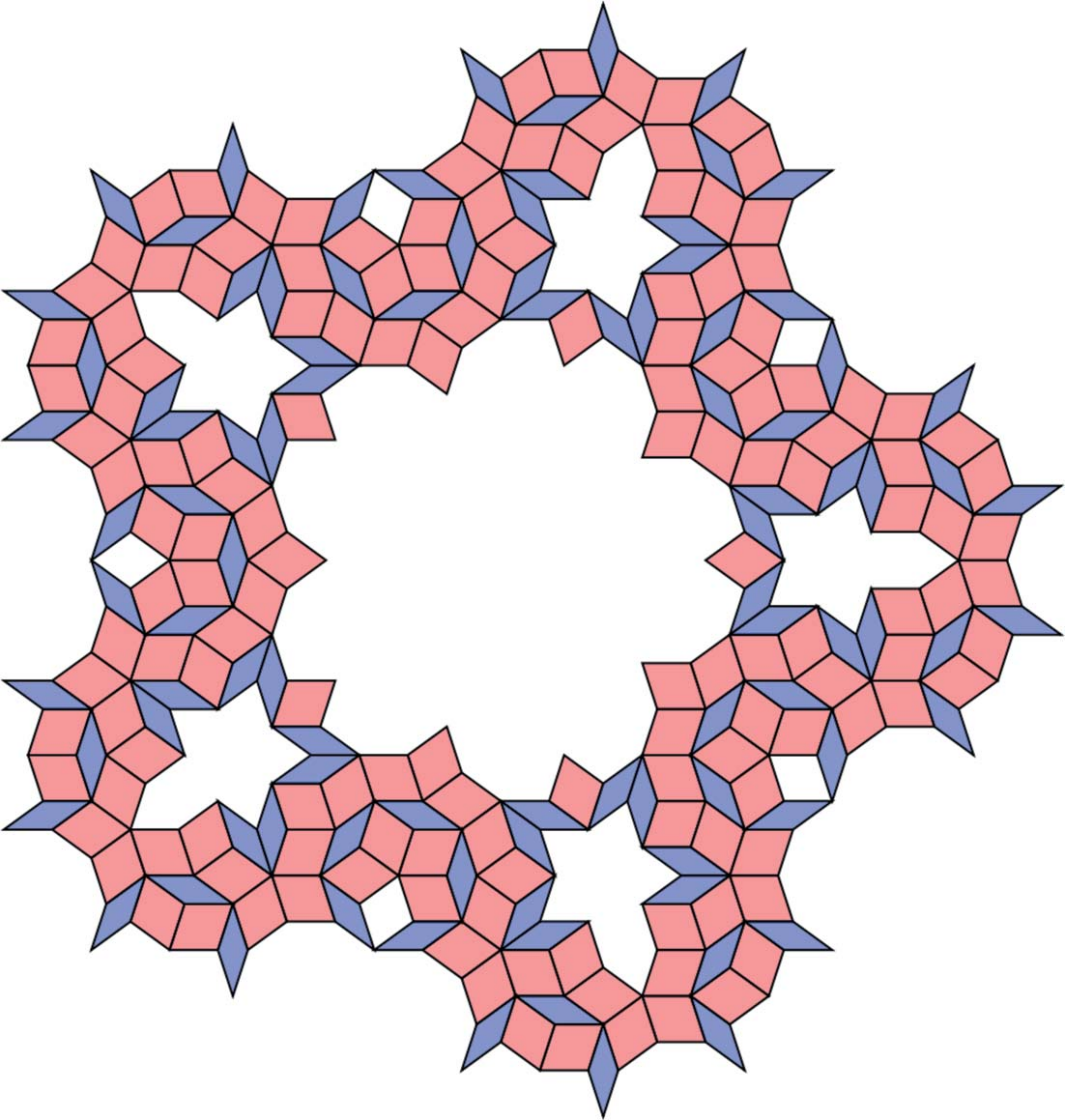
A 245-tile patch.

**Figure 17:**
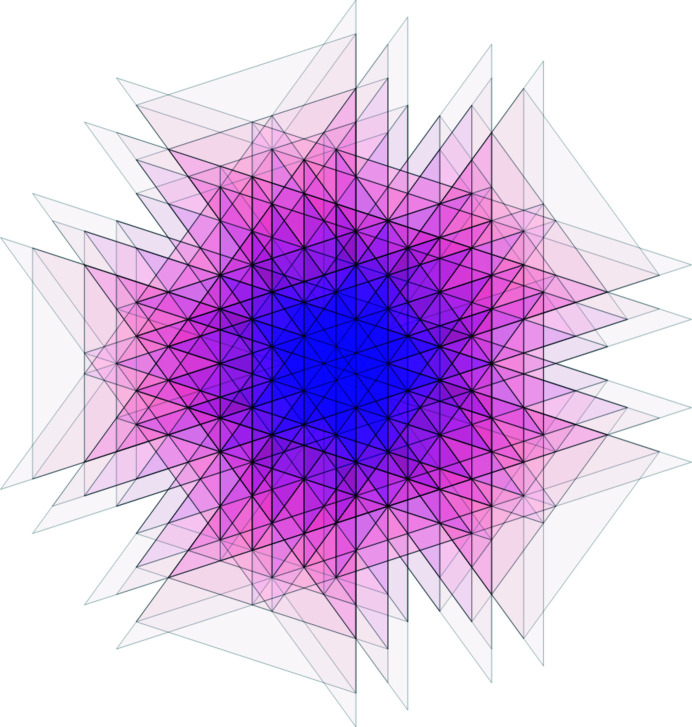
The dual image of the patch from Fig. 16. The frequency of this patch is 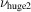 = 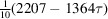 = 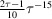 .

**Figure 18:**
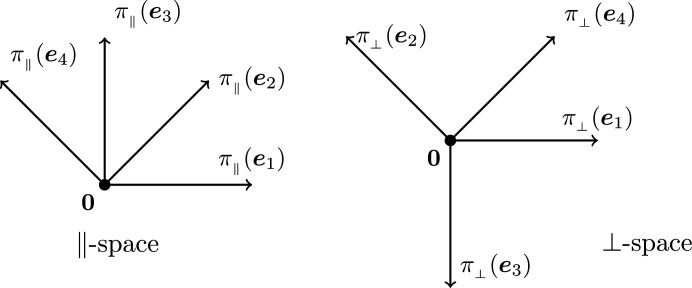
Projections of the standard basis **e**_1_, …, **e**_4_ onto the two subspaces.

**Figure 19:**
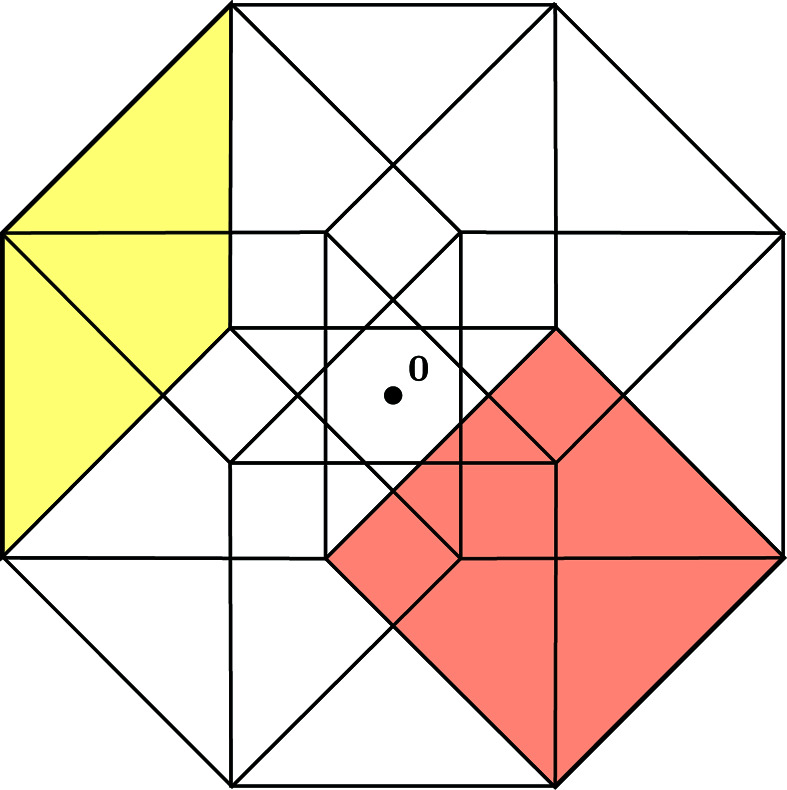
A projection of the Voronoi cell 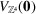 into the ⊥-space with two 2-boundaries indicated. The yellow rhombus corresponds to 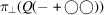 and the red square to 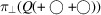 . In contrast with the Penrose tiling, the centre of the window is placed at the origin.

**Figure 20:**
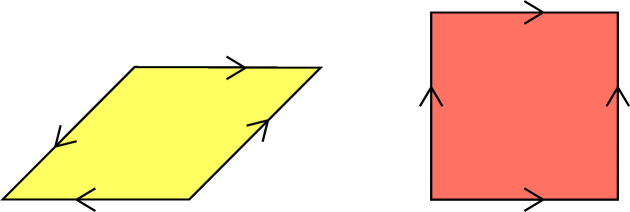
The decorated tiles for the Ammann–Beenker tiling. There are four different translation-equivalent rhombus tiles and eight different square tiles. They differ by a rotation by an integer multiple of 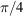 . In the case of the rhombus tiles, one has to decide on suitable representatives since the decorated tile possesses a rotation symmetry by π. We decided to pick up as the representatives the rhombus in the picture and its rotations by 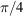 , 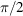 and 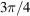 .

**Figure 21:**
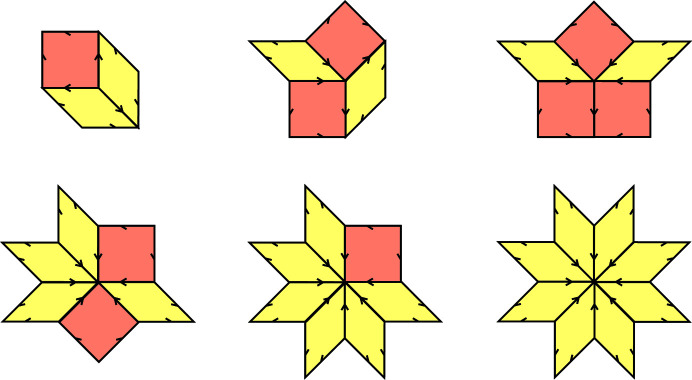
All allowed vertex configurations (up to rotations) within the Ammann–Beenker tiling displayed with decorations.

**Figure 22:**
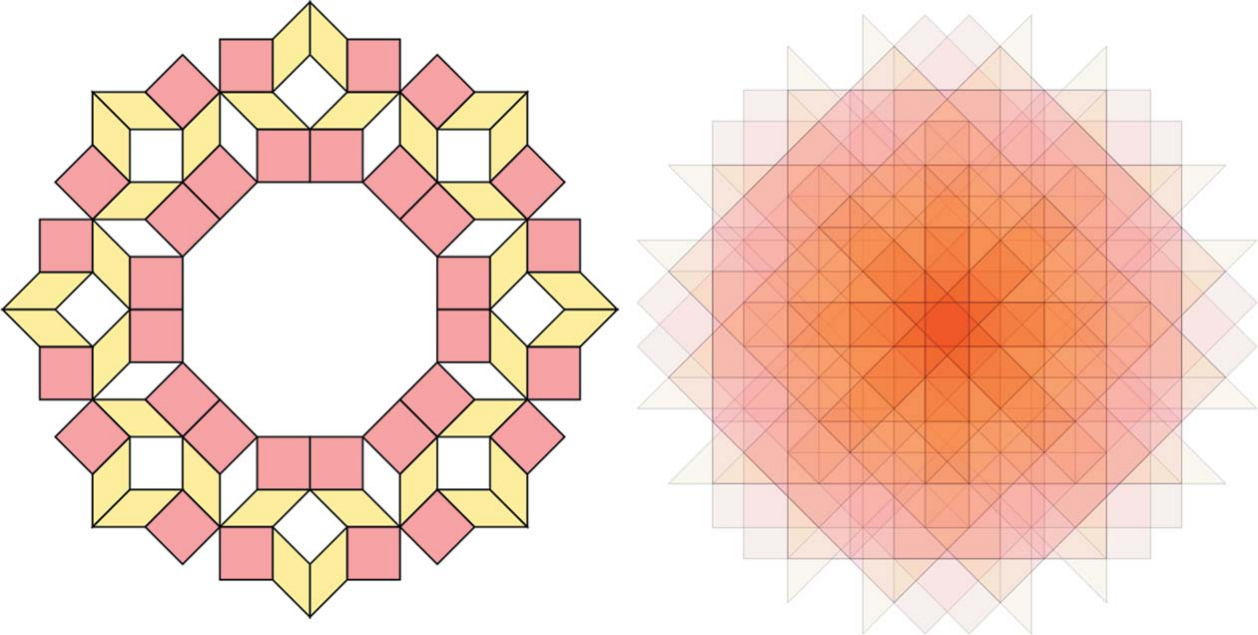
A 64-tile patch. Its frequency is ν_64_ = 29λ − 70 = λ^−5^.

**Figure 23:**
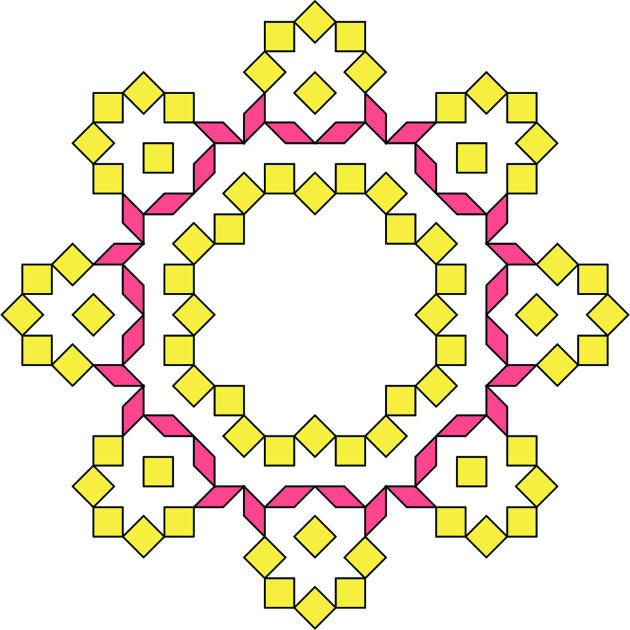
A 104-tile patch. Its frequency is ν_104_ = 985 − 408λ = λ^−8^.

**Figure 24:**
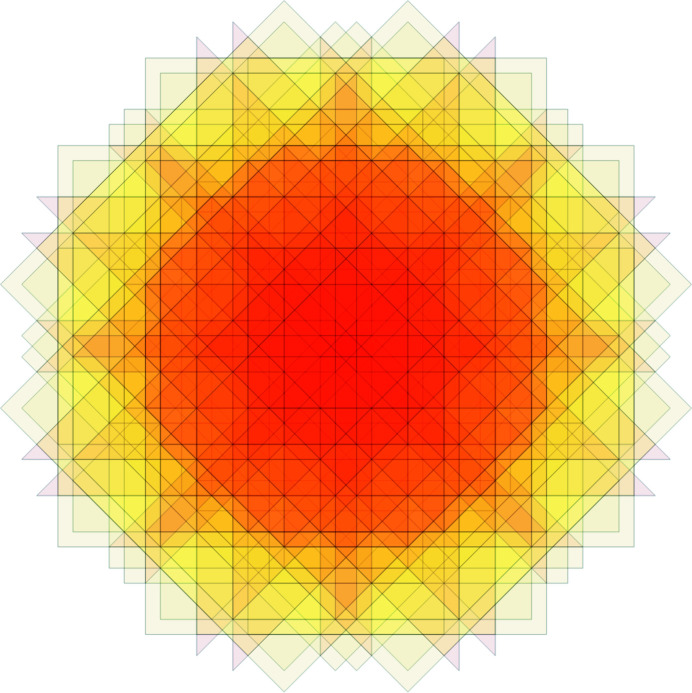
The intersection of dual tiles of the patch from Fig. 23.

**Figure 25:**
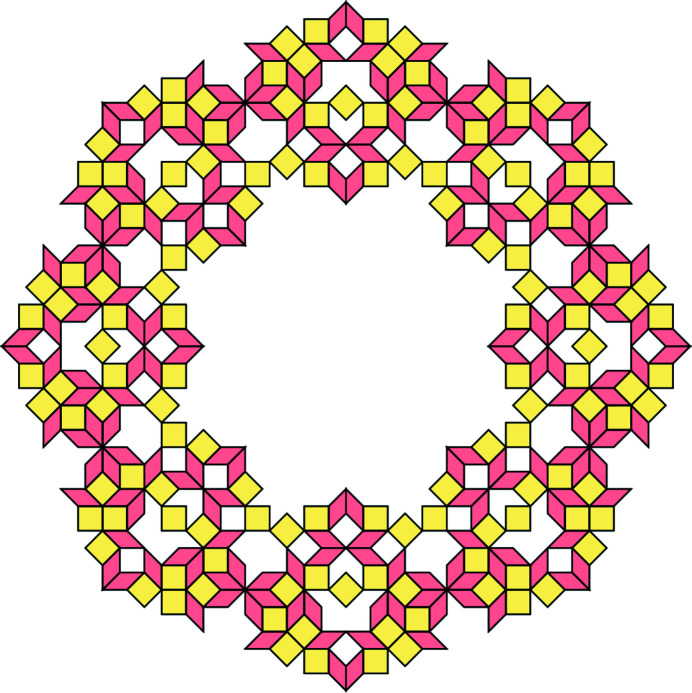
A 328-tile patch. Its frequency is ν_328_ = 985 − 408λ = λ^−8^.

**Figure 26:**
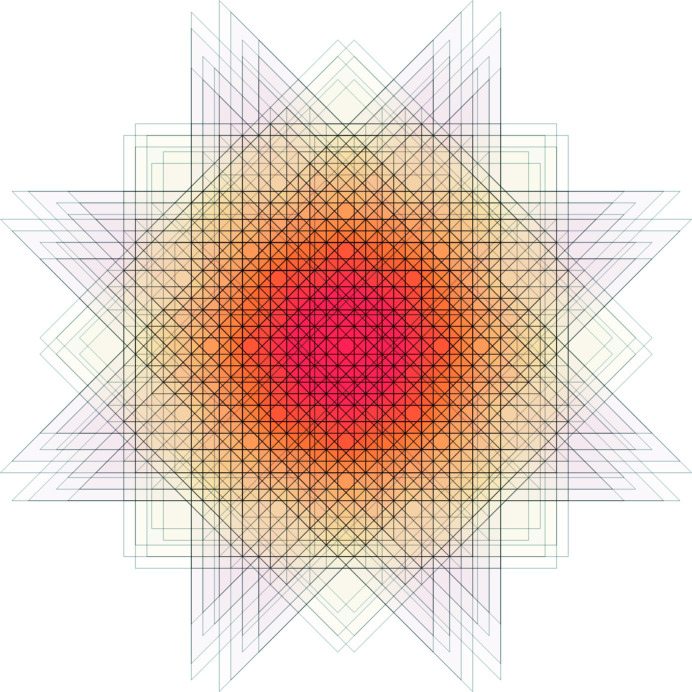
The intersection of dual tiles of the patch from Fig. 25.

**Table 1:** Frequencies of vertex configurations in Penrose tilings, all belonging to 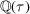 with τ being the golden ratio The second column shows the frequencies of particular patches, those in Fig. 7 ▸. The last column gives the total frequencies of a patch of a given type, *i.e.* a patch and all its images under the allowed rotations and space inversion.

Vertex configuration	Frequency 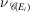	Total frequency ν_*i*_
1	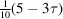	5 − 3τ = τ^−4^
2	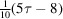	5τ − 8 = τ^−5^
3	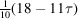	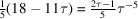
4	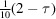	2 − τ = τ^−2^
5	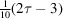	2τ − 3 = τ^−3^
6	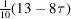	13 − 8τ = τ^−6^
7	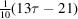	13τ − 21 = τ^−7^
8	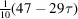	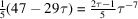

**Table 2:** The positions of shallow holes of the diamond ring patch relative to the origin *A*; in particular, this is the result of Step 4 of the algorithm For better readability, we abbreviate π_⊥_(**a**_*i*_) to 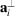 .

Shallow hole	Translation vector
*B*	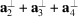
*C*	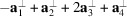
*D*	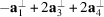
*E*	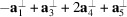
*F*	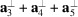
*G*	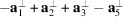
*H*	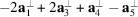
*I*	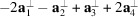
*J*	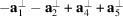

## Appendix A Exact results for patches in Penrose tilings

In Figs. 11 ▸ to 17 ▸ ▸ ▸ ▸ ▸ ▸, we depict other patches that appear in Damanik *et al.* (2022 ▸) and the corresponding dual triangles in  . We also give the frequencies of these patches.

## Appendix B Ammann–Beenker tiling

Here, we briefly describe the setting for the Ammann–Beenker octagonal tiling. This tiling can be obtained via the dualization of the four-dimensional cubic lattice  which is self-dual. Recall that the Voronoi cell around the origin is the 4-cube given as

The dual cells of the corresponding Voronoi complex are of the form

The symmetry group of the Voronoi cell is the *hyperoctahedral group* Ω(4) (Baake *et al.*, 1982 ▸). All 2-boundaries of  are squares of the form

and all its possible images under the action of Ω(4), which acts via permutations and sign flips. Altogether we obtain 24 congruent 2-boundaries. The dual boundaries are squares as well,

and the pairing of boundaries *Q* and their dual boundaries *Q** is one to one.

As in the case of the Penrose tiling, we need to find a suitable subgroup of the holohedry Ω(4) which possesses an (irreducible) representation in a plane. One can consider the dihedral group *D*_8_ which is a proper subgroup of Ω(4). This subgroup is generated by two elements *g*_8_, *s* satisfying  and (*g*_8_ *s*)^2^ = *e*. The generators act on the basis vectors **e**_*i*_ via the matrices

These matrices can be simultaneously brought to the real Jordan form, namely

using the matrix

Taking the first two entries of each column of *J*, one gains the projections of the basis vectors into the ∥-space, whereas taking the third and fourth ones gives their ⊥-projection. The projections are shown in Fig. 18 ▸ and they already reveal the two shapes of tiles, namely a square, and a rhombus with acute angle  .

The projections of the basis exhibit the desired octagonal symmetry. As above, we can project the 2-boundaries and obtain the Ammann–Beenker tiling as

We choose the vector **c**_⊥_ so that it does not belong to any 1-boundary of any Voronoi cell, similar to the Penrose case. In contrast with the Penrose tiling, we project the dual boundaries into the ∥-space, but this does not cause any difficulties. The ⊥-projection of the Voronoi cell with projections of two particular 2-boundaries is shown in Fig. 19 ▸. The area of the projection (which is an octagon) is  . Up to a translation, we have twelve different tiles – four rhombi and eight squares(!). This, perhaps surprising, fact follows from the decorations of the Ammann–Beenker tiles [see Baake & Grimm (2013 ▸) for further details]. Fig. 20 ▸ shows the tiles and their decorations.

As in the case of the Penrose tiling, we can determine all elementary polygons and obtain all possible vertex configurations as shown in Fig. 21 ▸.

Since there are no holes in this setting (as  is self-dual as a lattice), the algorithm for determining the patch frequencies has to be modified as follows. One has to replace ‘distinguishing between deep and shallow holes’ in Step 1 with ‘decorating the tiles’, and in Step 3 one has to replace ‘any shallow hole’ with ‘any vertex point’, since there is only a single translation class. And, of course, in the last step, one has to divide by the accurate area of the window, in this case by  . No other changes are needed. The patch frequencies are contained in the frequency module  which reads  with λ =  , the *silver mean* (Baake & Grimm, 2013 ▸, Example 7.9).

Figs. 22 ▸ to 26 ▸ ▸ ▸ ▸ show several patches of the Ammann–Beenker tiling which appear in Damanik *et al.* (2022 ▸), with their frequencies.
